# Effects of hypercholesterolism on expansion of abdominal aortic aneurysm in rat model

**DOI:** 10.1186/s13019-021-01734-1

**Published:** 2021-12-27

**Authors:** Jong Seok Lee, Sun Cheol Park, Sang Dong Kim

**Affiliations:** 1 Division of Vascular and Transplant Surgery, Department of Surgery, College of Medicine, The Catholic University of Korea, Seoul, 06591 Korea; 2 Division of Vascular and Transplant Surgery, Department of Surgery, Incheon St. Mary’s Hospital, The Catholic University of Korea, 56 Dongsu-ro, Bupyong-Gu, Incheon, 21431 Korea

**Keywords:** Abdominal aortic aneurysm, Animal model, Hypercholesterolemia, Statin

## Abstract

**Background:**

Inflammation is recognized as a critical process in expansion of abdominal aortic aneurysm (AAA). A relationship between effects of cholesterol and statin in this process have been suggested, but remain untested. Therefore, current study aimed to examine the effects of hypercholesterolism on expansion of AAA in a rat model.

**Methods:**

A total of 16 male rats were divided into 4 groups as follows: group I, normocholesterol diet and saline infusion, group II, normocholesterol diet and porcine pancreatic elastase (PPE) infusion, group III, hypercholesterol diet and PPE infusion, and group IV, hypercholesterol diet, PPE infusion and statin administration. At the 3rd week, saline was infused intraluminally in group I and PPE in groups II-IV to induce AAA. At the 5th week, blood and aortic tissue were obtained from each rat for evaluation of lipid profiles, aortic diameters (ADs), and characteristics of stains.

**Results:**

Post-procedural aortic diameter (AD3) and AD3/pre-procedural aortic diameter (AD1) were significantly different among four groups (P = 0.042, P = 0.028, respectively). AD3 was significantly larger in group II than group I, and group III than group IV (P = 0.012, P = 0.043, respectively). AD3/AD1 was significantly higher in group II than group I, and group III than group II (P = 0.008, P = 0.030, respectively). Group III showed the highest cellularity for inflammatory cells.

**Conclusions:**

Though larger experimental and clinical studies are necessary, authors suggest that hypercholesterolism can aggravate expansion of AAA, and that statin therapy can reduce it. Therefore, monitoring for hypercholesterolism and instituting statin therapy may be helpful to suppress expansion of AAA.

## Background

Abdominal aortic aneurysm (AAA) has a complex pathogenesis and is affected by a variety of risk factors, including age, gender, smoking, hypertension, family history and atherosclerosis [1, 2]. Atherosclerosis is a degenerative process mediated by modification of low-density lipoproteins (LDL) and unregulated uptake by macrophages within the artery wall. This process appears to be associated with AAA, though the underlying pathophysiology of the aortic dilation process is not well understood [2, 3]. Recently, AAA development has been suggested as part of a significant and dynamic remodeling process in the aortic wall characterized by the depletion of smooth muscle cells (SMCs), chronic medial and adventitial inflammation, elastin degeneration and medial attenuation [2, 4, 5]. The reduction of vascular inflammation can be shown to address a key pathophysiologic collagenolytic pathway involved in AAA progression [6]. Several studies have suggested that statins can reduce vascular inflammation and can stimulate atherosclerotic plaque regression [6–8]. Unfortunately, there are no pharmacotherapies proven to attenuate the rate of progression and minimize the rupture risk of AAA [6]. Given that more than 60% of patients with small AAA were treated surgically due to aneurysm enlargement or signs of rupture during follow-up, there is an urgent need to understand the development of AAA, and to identify appropriate medical therapy using drugs targeting the progressive expansion of AAA [9]. Therefore, we evaluated the effects of hypercholesterolism and use of a lipid lowering agent (statin) on the expansion of AAA in a rat model.

## Materials and methods

### Experimental animals

A total of 16 male Sprague–Dawley (SD) rats that were six weeks of age and weighed 180-200 g, were included. Rats were randomly divided into 4 groups (4 rats in each group): group I (control diet, saline infused, control water), group II (control diet, porcine pancreatic elastase (PPE) infused, control water), group III (hypercholesterol diet, PPE infused, control water), and group IV (hypercholesterol diet, PPE infused, water mixed with statin) [10, 11]. The control diet was both low fat and low cholesterol (D12337) (Research Diets, New Brunswick, NJ, USA), while the hypercholesterol diet was a rat chow designed to be high in cholesterol (Paigen’s atherogenic rodent diet, D12336) (Research Diets, New Brunswick, NJ, USA) [10]. Atorvastatin (Lipitor, Pfizer, NY, USA) (0.2 μg/g body weight/day) was administered by standard oral gavage in group IV for 5 weeks [1].

### Surgical Procedures

**Fig. 1:**
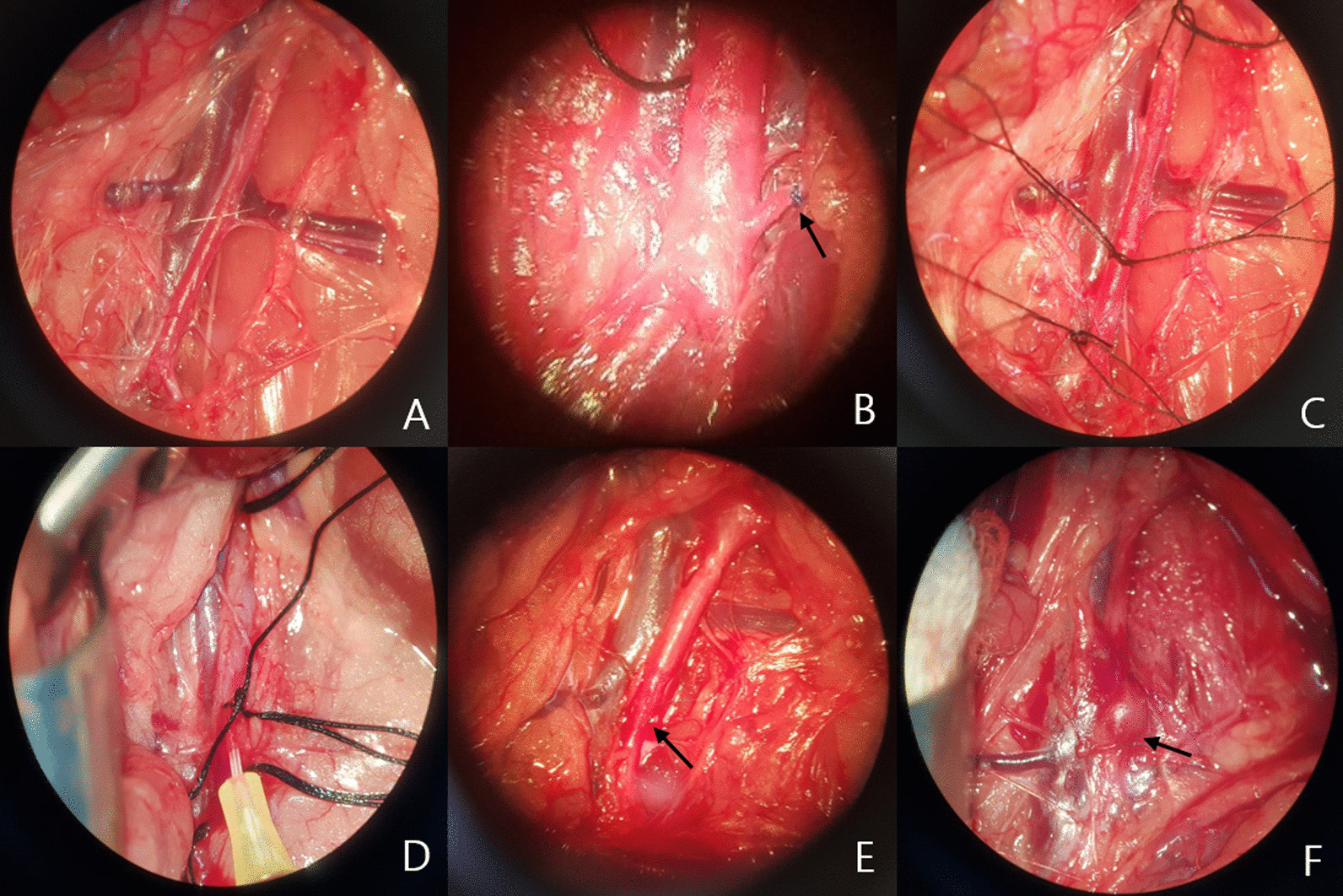
Procedures in AAA model. **A** Aortic dissection, **B** Ligation of aortic branches, **C** Aortic isolation, **D** PPE injection, **E** Recovered blood flow, and **F** AAA induction. Arrows indicate ligated artery, repaired aorta, and AAA in **B**, **E**, **F**, respectively

During the third week, rats were anesthetized using 2% inhaled isoflurane, and surgery was carried out by a vascular surgeon as follows [11–13]. Periaortic dissection was performed from renal vein level to bifurcation of aorta (Fig. 1A). The initial aortic diameter (AD1) was measured with a micrometer caliper in all groups. Aortic branches such as lumbar arteries were ligated with suture (polypropylene 8-0) (Prolene, J&J, NJ, USA) (Fig. 1B). Three silk sutures (4-0, Ethicon, J&J, NJ, USA) were separately prepared for three ties at the renal vein level, incision point, and bifurcation of aorta (Fig. 1C). At first, two ties at renal vein level and bifurcation of aorta were performed to minimize procedural bleeding. The initial ties were followed with a small incision placed above a tie at the bifurcation site of the aorta, followed by insertion of the angiocatheter (26G, Polypen, Poly Medicure, Brussels, Belgium). The third tie of aorta was performed to fix the angiocatheter in place and minimize bleeding between the angiocatheter and the incision of aorta (Fig. 1D). The isolated region of the aorta was then injected with saline in group I or type I PPE (30 U/ml) (5.9 U/mg; Sigma Aldrich, St. Louis, MO, USA) in group II-IV [11, 12]. Using 10 ml of saline in group I or a 10 ml saline solution mixed with 3 ml PPE in group II-IV, approximately 1 ml of solution was loaded in the aorta for 5 min with an infusion pump operated at 2 atmospheric pressure [9, 11, 12]. During this period, expanded aortic diameter (AD2) was measured with a micrometer caliper in all groups. All ties were removed along with the angiocatheter after successful completion of the perfusion, followed by closure of the incision using a suture (polypropylene 8-0) (Prolene, J&J, NJ, USA) [11, 12]. Recovery of blood flow in the aorta and common iliac artery (Fig. 1E) were performed and ischemic time and procedural time were checked. Ischemic time is equal to aorta clamping time in the current study.

At the 5th week, aneurysmal change was inspected (Fig. 1F) and the final aortic diameter (AD3) was measured using a micrometer caliper after aortic dissection via laparotomy under anesthesia using 2% inhaled isoflurane. A 1 cm-sized infrarenal segment of aorta and 1 ml blood was obtained from each rat. Rats were euthanized with carbon dioxide according to the guidelines of our institute [10].

### Laboratory and histologic analysis

Body weights of all rats were measured at the first and fifth week. Total cholesterol (TC), high density lipoprotein (HDL), low density lipoprotein (LDL), and triglyceride (TG) levels were evaluated in blood samples from each rat. Hematoxylin and eosin (H&E) staining, and immunohistochemistry (IH) with alpha smooth muscle actin (α-SMA) antibody were carried out on embedded aortic tissue sections cut following fixation in 10% formalin solution [10]. The degree of cellularity was assessed at a magnification of 400 (× 400) using a magnified-high power field (HPF) microscope. The number of inflammatory cells in media and adventitia was measured twice by examiners blind to group assignments [10].

### Immunohistochemistry (IH) with alpha smooth muscle actin (α-SMA) antibody

For detection of α-SMA in tissues, tissue sections were incubated with 0.5% TritonX-100 (Sigma Aldrich, St. Louis, MO, USA) in 0.01 M phosphate-buffered saline (PBS) and blocked with normal donkey serum [10]. Subsequently, the tissue sections were incubated overnight at 4 °C with primary antibodies against α-SMA [10]. Sections were rinsed in PBS and incubated in peroxidase-conjugated donkey anti-mouse or rabbit antimouse IgG (Amersham Pharmacia Biotech, Piscataway, NJ, USA) [10]. Following a rinse in Tris-buffered saline (TBS) (Sigma Aldrich, St. Louis, MO, USA), the tissue sections were incubated with a mixture of 0.05% 3,3-diaminobenzidine until a brown color was visible, washed with TBS, counterstained with hematoxylin, and examined by light microscopy [10].

### Statistical analysis

Statistical analysis included Student t-test, Fisher's exact test, Wilcoxon test, and ANOVA using SPSS 20.0 (IBM Inc., Armonk, NY, USA). A P value of less than 0.05 was considered statistically significant. Data are presented as mean ± standard deviation.

This research was approved by the Institutional Animal Care and Use Committee (IACUC) of our institute (CIMH-2019-020).

## Results

**Fig. 2:**
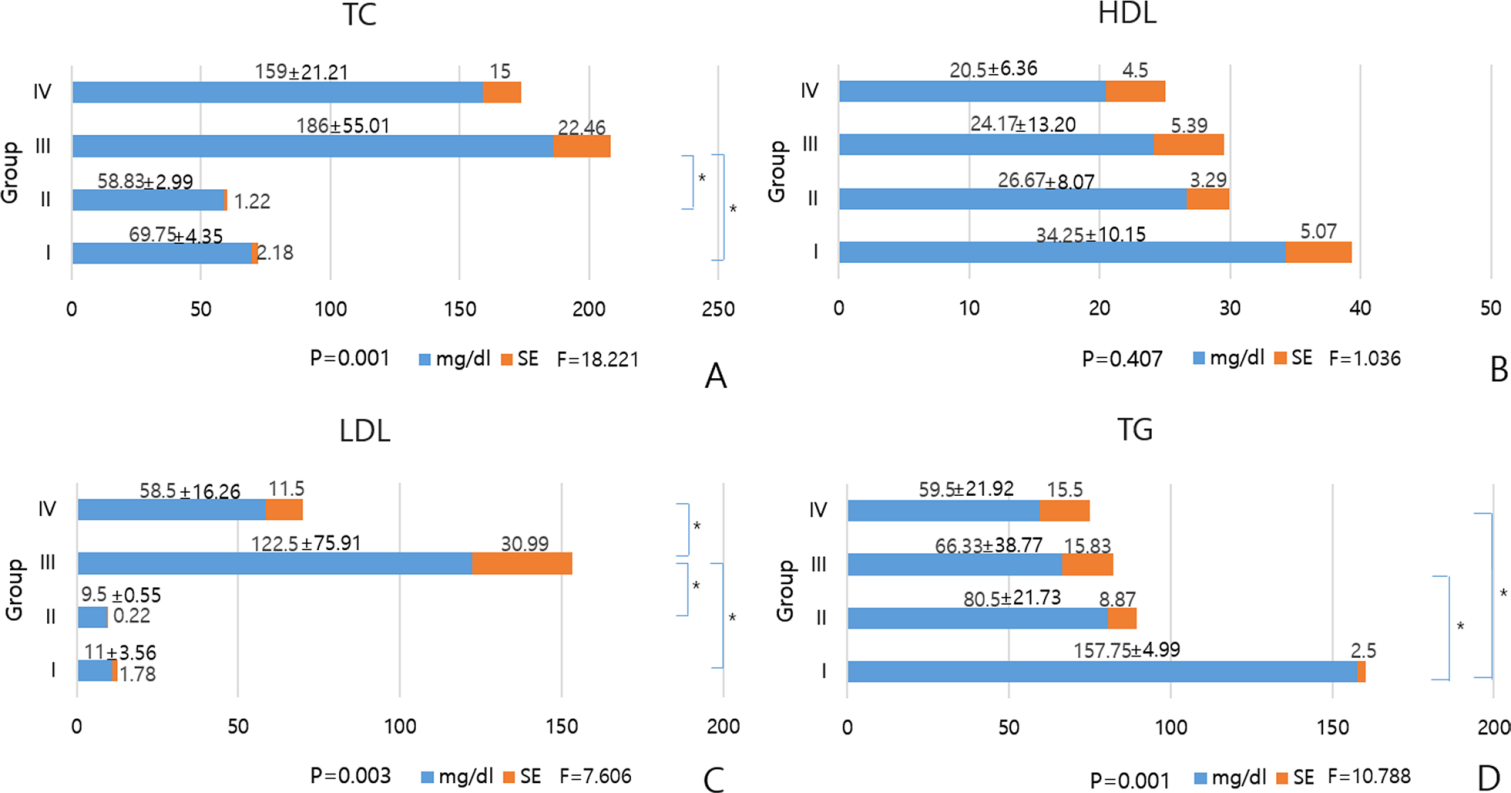
Differences in laboratory testing between groups. **A** Total cholesterol (TC), **B** High density lipoprotein (HDL), **C** Low density lipoprotein (LDL), and **D** Triglyceride (TG). *P < 0.05, SE = standard error, F = Fisher's exact test

Three rats were lost during surgical procedures, one due to massive bleeding and two postoperatively for unknown causes. Lost three rats consisted of one from group I, one from group III, and one from group IV. Finally, survived 13 rats were included in three in group I, four in group II, three in group III, and three in group IV. Among lost three rats, one from group I was lost by massive bleeding, and one from group III and one from group IV were lost by unknown causes. The remaining 13 rats were completed all remaining stages of the experimental procedures without incident. The lipid profiles for TC, LDL, and TG were significantly different among the four groups (P = 0.001) (Fig. 2). TC (mg/dl) was significantly higher in group III compared to group I (186.00 ± 55.01 vs 69.75 ± 4.35; P = 0.003), and significantly higher in group III compared to group II (186.00 ± 55.01 vs 58.83 ± 2.99; P = 0.002) (Fig. 2). LDL (mg/dl) was significantly higher in group III compared to group I (122.5 ± 75.91 vs 11.00 ± 3.56; P = 0.015), significantly higher in group III compared to group II (122.5 ± 75.91 vs 9.50 ± 0.55; P = 0.015), and significantly higher in group III compared to group IV (122.5 ± 75.91 vs 58.50 ± 16.26; P = 0.033) (Fig. 2). TG (mg/dl) was significantly higher in group I compared to group III (157.75 ± 4.99 vs 66.33 ± 38.77; P = 0.002), and significantly higher in group I compared to group IV (157.75 ± 4.99 vs 59.5 ± 21.92; P = 0.009) (Fig. 2). HDL was not significantly different between groups (P > 0.05) (Fig. 2).

In terms of ordinal series comparison with rank (R) cases, TC (R), LDL (R), and TG (R) were significantly different among the four groups (P = 0.001, P = 0.002, P = 0.038, respectively). TC (R) was significantly higher in group III compared to group I (10.67 ± 2.08 vs 6.00 ± 1.00; P = 0.025), and significantly higher in group III compared to group II (10.67 ± 2.08 vs 2.5 ± 1.22; P = 0.001). LDL (R) was significantly higher in group III compared to group I (11.33 ± 2.08 vs 4.67 ± 3.21; P = 0.039), and significantly higher in group IV compared to group II (9.67 ± 1.53 vs 3.50 ± 1.15; P = 0.002). TG (R) was significantly higher in group I compared to group II (12.00 ± 1.00 vs 6.25 ± 2.60; P = 0.016), and significantly higher in group I compared to group IV (12.00 ± 1.00 vs 4.00 ± 2.65; P = 0.008).

**Fig. 3:**
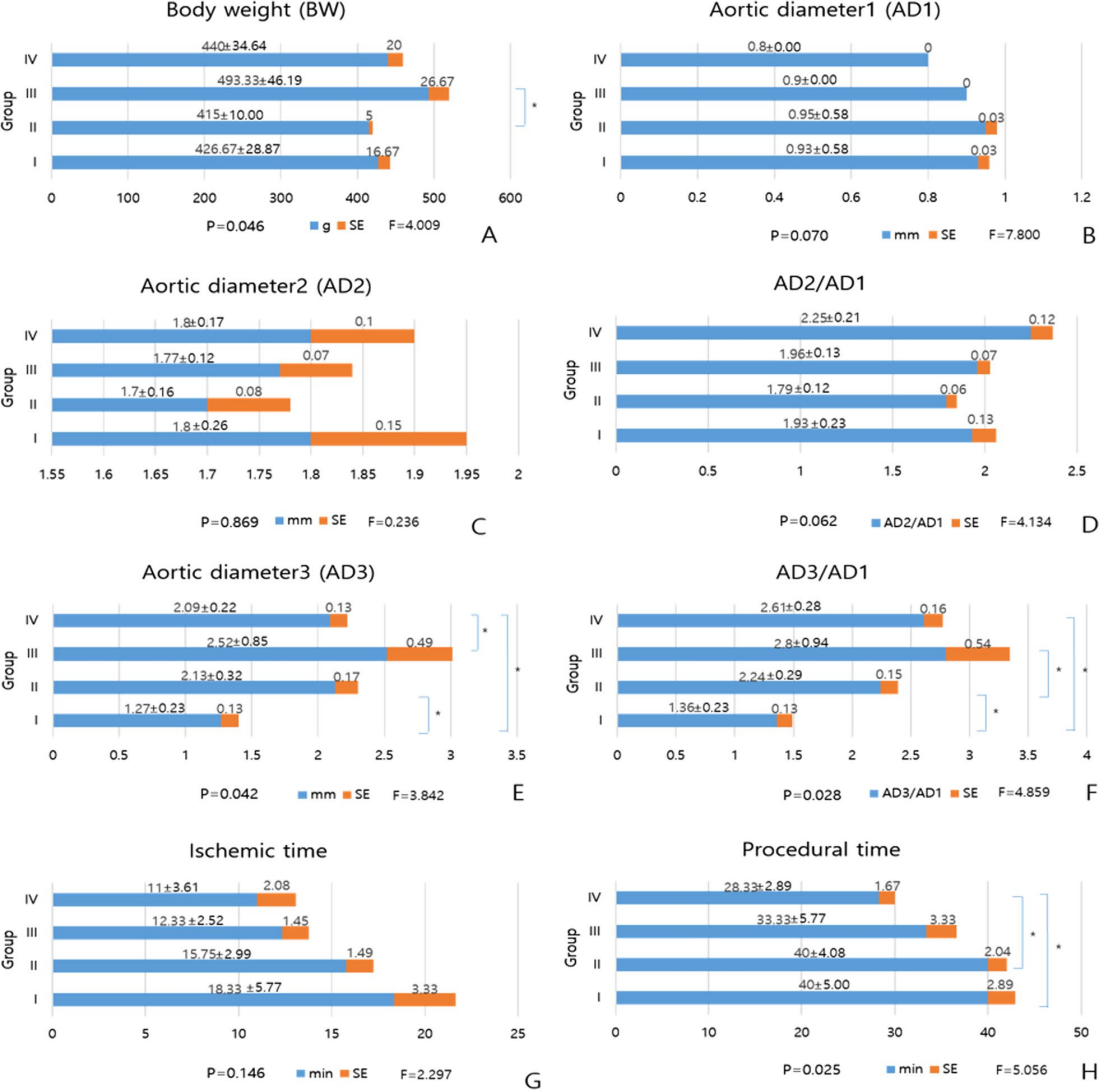
Differences in procedural factors. **A** Body weight, **B** AD1, **C** AD2, **D** Ratio of AD2 to AD1, **E** AD3, **F** Ratio of AD3 to AD1, **G** Ischemic time, and **H** Procedural time. *P < 0.05, SE = standard error, F = Fisher's exact test

Body weight at the fifth week was significantly larger in group III compared to group II (493.33 ± 46.19 vs 415.00 ± 10.00; P = 0.037) (Fig. 3). AD1, AD2, and AD2/AD1 were not significantly different among the four groups (P > 0.05), with measures of 0.90 ± 0.71 mm, 1.76 ± 0.17 mm, and 1.97 ± 0.23, respectively (Fig. 3). AD3 and AD3/AD1 were significantly different between the four groups (P = 0.042 and 0.028) (Fig. 3). AD3 was significantly larger in group II compared to group I (2.13 ± 0.32 vs 1.27 ± 0.23; P = 0.012), significantly larger in group III compared to group IV (2.52 ± 0.85 vs 2.09 ± 0.22; P = 0.043), and significantly larger in group IV compared to group I (2.09 ± 0.22 vs 1.27 ± 0.23; P = 0.010) (Fig. 3). AD3/AD1 was significantly higher in group II compared to group I (2.24 ± 0.29 vs 1.36 ± 0.23; P = 0.008), significantly higher in group III compared to group II (2.80 ± 0.94 vs 2.24 ± 0.29; P = 0.030), and significantly higher in group IV compared to group I (2.61 ± 0.28 vs 1.36 ± 0.23; P = 0.004) (Fig. 3). Ischemic time was not significantly different between the four groups (P > 0.05), with a mean measurement of 14.46 ± 4.41 min (Fig. 3). Procedural time (PT) was significantly different among four groups (P = 0.025) (Fig. 3), with significantly larger times in group II compared to group IV (40.00 ± 4.08 vs 28.33 ± 5.77; P = 0.010), significantly larger in group I compared to group IV (40.00 ± 5.00 vs 28.33 ± 5.77; P = 0.020), with a mean value of 35.77 ± 6.41 min overall (Fig. 3).

In terms of ordinal series comparison with rank cases, AD3 (R), AD3/AD1 (R), and PT (R) were significantly different among the four groups (P = 0.044, P = 0.015, P = 0.026, respectively). AD3 (R) was significantly higher in group II compared to group I (8.00 ± 3.16 vs 2.00 ± 0.87; P = 0.026), significantly higher in group III compared to group I (9.67 ± 3.06 vs 2.00 ± 0.87; P = 0.014), and significantly higher in group IV compared to group I (8.00 ± 3.61 vs 2.00 ± 0.87; P = 0.049). AD3/AD1 (R) was significantly higher in group II compared to group I (6.75 ± 2.75 vs 2.00 ± 1.00; P = 0.034), significantly higher in group III compared to group I (9.00 ± 3.61 vs 2.00 ± 1.00; P = 0.032), and significantly higher in group IV compared to group I (10.33 ± 2.08 vs 2.00 ± 1.00; P = 0.003). PT (R) was significantly higher in group I compared to group IV (9.50 ± 3.00 vs 2.67 ± 1.44; P = 0.024), and significantly higher in group II compared to group IV (9.50 ± 2.45 vs 2.67 ± 1.44; P = 0.008).

**Fig. 4:**
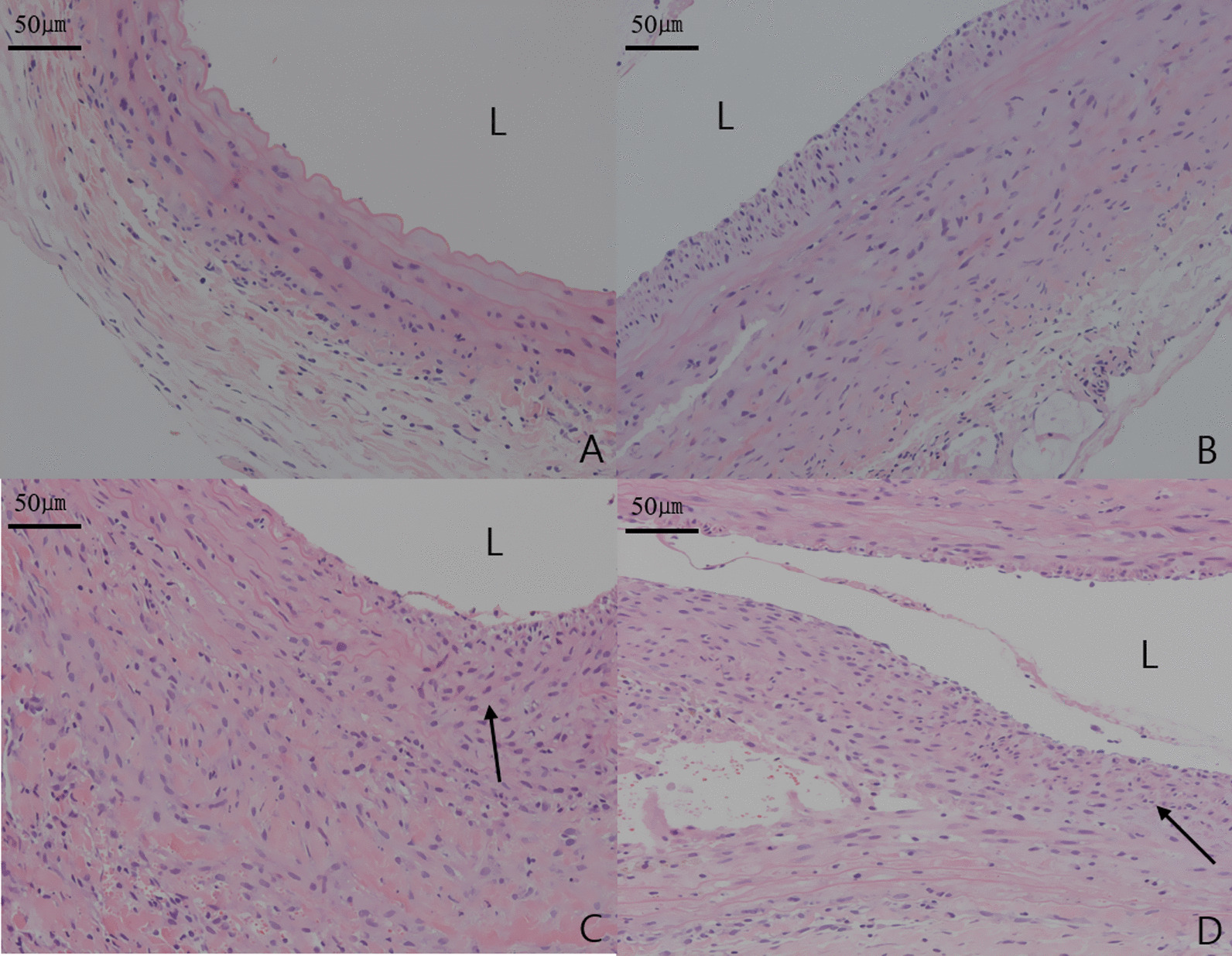
H&E stains (× 200) show differences among groups. **A** aorta of rats included in group I, **B** group II, **C** group III, and **D** group IV. Arrows indicate the infiltration of inflammatory cells in **C** and **D**

**Fig. 5:**
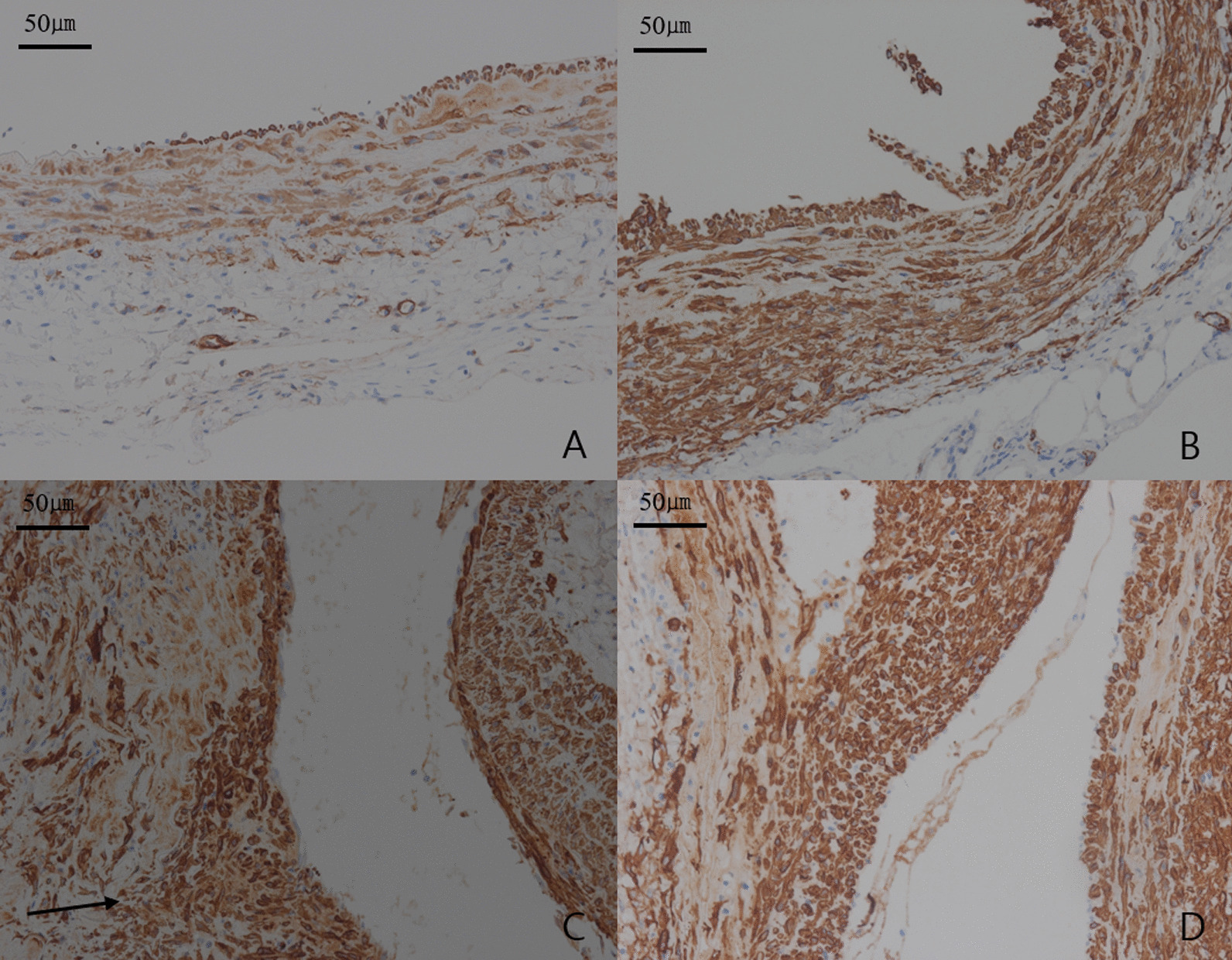
Immunohistochemistry with anti-α-SMA antibody (× 200) of aorta show differences among groups. **A** aorta of rats included in group I, **B** group II, **C** group III, and **D** group IV. An arrow indicates the migration of smooth muscle cells

Analysis of H&E staining results, indicated a remodeling process characterized by degeneration of the extracellular matrix (ECM), depletion of SMCs, medial attenuation, and infiltration of inflammatory cells into the adventitial and medial layers [2, 11], that was more pronounced in group II than group I (Fig. 4). Among PPE injected groups (group II-IV), group III had the most remarkable findings, indicating a remodeling process for AAA (Fig. 4). According to IH with α-SMA antibody staining findings, migration and proliferation of SMCs had greater expression in group II than group I (Fig. 5). Among PPE injected groups, group II showed the greatest expression of migration and proliferation of SMCs, while depletion of SMCs was the most prominent in group III (Fig. 5).

According to H&E staining findings, the inflammatory cellularity in media and adventitia, assessed at a magnification of × 400, revealed significant differences between the four groups (P < 0.05). The number of inflammatory cells in media and adventitia per each view was significantly larger in group II compared to group I (232.50 ± 27.54 vs 128.00 ± 17.01; P = 0.002), larger in group III compared to group II (298.33 ± 10.41 vs 232.50 ± 27.54; P = 0.012), and larger in group III compared to group IV (298.33 ± 10.41 vs 260,00 ± 10.00; P = 0.010). However, there was no significantly difference between group II and group IV (232.50 ± 27.54 vs 260.00 ± 10.00; P = 0.140), with an overall mean of 229.92 ± 65.33. The inflammatory cellularity in media and adventitia was greater in PPE injected groups than group I. Among PPE injected groups, group III showed the greatest cellularity for inflammatory cells. Also, according to IH with α-SMA antibody staining findings, the SMCs in media and adventitia, assessed at a magnification of × 400, revealed significant differences between the four groups (P < 0.05). The number of SMCs in media and adventitia per each view was significantly larger in group II compared to group I (228.25 ± 12.87 vs 77.00 ± 9.85; P = 0.001), larger in group II compared to group III (228.25 ± 12.87 vs 136.67 ± 23.63; P = 0.001), and larger in group II compared to group IV (228.25 ± 12.87 vs 176.67 ± 15.28; P = 0.005). However, there was no significantly difference between group III and group IV (136.67 ± 23.63 vs 176.67 ± 15.28; P = 0.070), with an overall mean of 160.31 ± 60.58. The SMCs in media and adventitia were much more in PPE injected groups than group I. Among PPE injected groups, group II showed the greatest cellularity for SMCs, and group III showed prominent depletion of SMCs.

## Discussion

Sudden death resulting from rupture of an aneurysm is strongly related to AAA [13]. Although the introduction of endovascular abdominal aortic repair (EVAR) has significantly reduced the risk of periprocedural morbidity and mortality, the identification and validation of effective medical therapies to suppress progression of early AAA remains a significant unmet medical need [14]. For patients where repair is not indicated, the current recommendation is clinical follow-up with observations performed regularly to limit the progression of AAA [9, 15]. Unfortunately, even though most AAAs are identified at an early stage of the disease, no medical intervention, including aggressive attempts to modify conventional cardiovascular risk factors, has proven effective in limiting progressive diameter enlargement or eventual rupture [14, 16]. Considering that patients with small AAA are often treated surgically due to aneurysm enlargement or signs of rupture during follow-up [9], there is an urgent need to understand the development of AAA and to identify drugs targeting the progressive expansion of the disease [9].

Animal models are useful strategic tools to investigate the mechanisms underlying the formation and development of AAAs [2, 13]. To date, several animal models including the elastase (PPE) model, the calcium chloride model, the angiotensin II model, the xenograft model, and the transgenic model of AAA have been introduced in rodents [2, 9, 13]. Among these models, the PPE model has been used most extensively due to high reproducibility in smaller animals and the occurrence of dilatation at 1 week following induction, though this model requires technically difficult major surgery [9, 11–13]. In the current study, we induced the PPE model of AAA in rats [9, 11, 12]. A comparison of control (group I) and PPE injected (group II) groups indicated that AD3 was significantly larger in group II compared with group I, and AD3/AD1 was significantly higher in group II compared to group I. These results suggest that the PPE model of AAA was effectively induced. TG was significantly higher in group I compared to group (II to IV). The difference between group I and group II to IV was whether PPE was applied or not. And, a report suggested that there could be possibility of correlation between lipid and cation contents and susceptibility to elastolysis [17]. So, we suggest that we need to evaluate an effect of PPE on TG through further study.

With recent deepening of research in vascular pathophysiology and molecular biology, pathological changes of AAA wall including vascular SMC apoptosis, oxidative stress, chronic inflammation, as well as over-degradation and remodeling of ECM have been described [2, 9, 18]. Medial degeneration with simultaneous destruction of SMC and elastic lamellae is a pathognomonic sign of human aneurysmatic aorta and is evident in all AAA induction studies using animal models [2, 9, 11]. Evidence suggests that the inflammatory process is essential for AAA formation in humans and that certain inflammatory mediators including matrix metalloproteinase (MMP)-2 and MMP-9, plays a key role [2]. The inflammatory reaction is important for the development and progression of AAA and stimulates effects including chronic inflammation, ECM deterioration, and vascular structure remodeling [11, 19]. This inflammatory process is evident in aorta harvested from animals subjected to chemical induction [2]. Pathophysiological research has focused on the impact of changes in the expression of elastin-degrading enzymes on human and experimental AAA, especially MMPs, thiol proteases, and their respective inhibitors [11]. Atherosclerosis is a common underlying pathophysiologic phenomenon in coronary, peripheral and aneurysmal disease, and plays a role in AAA development in humans. Similarly, certain induction methods in animals seem to activate the formation of atherosclerosis as well [2, 6]. Furthermore, atherosclerosis is related to increased intra-aortic thrombus formation, another process related to the development of AAA in humans, and macrophages have been found to participate in aortic diseases including atherosclerosis and AAA. Several histopathological features of AAA resemble those of atherosclerosis, including macrophage infiltration, cholesterol-loaded macrophage-derived foam cells, apoptotic macrophages, and SMC senescence [11]. In the current study, among PPE injected groups (group II to IV), AD3 was significantly larger in group III (hypercholesterol group) compared to group II (normocholesterol group), and AD3/AD1 was significantly higher in group III compared to group II. Similarly, inflammatory cellularity was significantly greater in group III compared to group II. Taken together, these results suggest that cholesterol was proportionally associated with inflammation of aortic wall and expansion of AAA.

Unfortunately, there are no current pharmacotherapies proven to attenuate the rate of progression and minimize the rupture risk of AAA [6]. Several studies have demonstrated that statins (lipid-lowering agents) reduce vascular inflammation and can stimulate atherosclerotic plaque regression [6–8]. The reduction of vascular inflammation has been shown to address a key pathophysiologic collagenolytic pathway involved in AAA progression [6, 19]. Furthermore, statins have been shown in both animal and human studies to reduce collagen breakdown by stabilizing imbalances in MMPs and tissue inhibitors of MMPs [6, 20]. Salata et al. [6], suggested that statin application would not only reduce the growth rate of AAA but could also reduce the risk of mortality in patients undergoing open repair perioperatively. Although the underlying mechanism has not been well understood thus far, statin application has exerted protective effects on the endothelial function, inflammatory reaction, oxidative stress, thrombosis and plaque stability, affecting the occurrence of cardiovascular complications and the progression of AAA [21, 22]. Luan et al. [23], demonstrated that statin application would alter the inflammatory environment of aneurysm inflammation, rather than directly lowering the blood lipid levels. Evidence both in vitro and in vivo found that statin therapy downregulates the expression of metalloproteinase [21, 23]. The current study found that between hypercholesterol groups (group III and IV), AD3 was significantly larger in group III (statin(-)) compared with group IV (statin(+)) and AD3/AD1 was higher in group III compared to group II although this difference did not reach statistical significance. Therefore, we suggest that a lipid-lowering agent (statin) plays a role in reducing the expansion of AAA.

There are several limitations. This study presents small sized samples, and provides preliminary data that must be confirmed through larger sampled follow-up experiments to identify the detailed mechanisms [11]. Any similarity between animal models and human aneurysms is based on tissue samples from end-stage disease [2]. Aneurysm formation in humans is a long process, and the underlying pathophysiological process behind it is mostly unknown [2]. This is presented by the fact that results from animal AAA-model studies have consistently been very difficult to be reproduced in humans [2]. The variable animal models used to induce AAAs indicates that no model completely mirrors the human AAA [13]. This is a central limitation with animal models and should be taken into consideration when evaluating the results from AAA animal models [2].

## Conclusion

Though larger experimental and clinical studies are necessary to confirm the effects of hypercholesterolism and statin therapy on the expansion of AAA, findings from the current study suggest that hypercholesterolism can proportionally expand AAA, which can be reduced by statin therapy. Therefore, monitoring and management such as statin therapy for hypercholesterolism may be helpful to prevent expansion of AAA.

## Data Availability

The datasets generated and analysed during the current study are available from the corresponding author on reasonable request.

## Abbreviations

AAA: Abdominal aortic aneurysm
α-SMA: Alpha smooth muscle actin
AD: Aortic diameter
ECM: Extracellular matrix
EVAR: Endovascular aortic aneurysmal repair
HDL: High density lipoprotein
H&E: Hematoxylin and eosin
HPF: High power field
IH: Immunohistochemistry
LDL: Low-density lipoprotein
MMP: Matrix metalloproteinase
PPE: Porcine pancreatic elastase
SD: Sprague–Dawley
SMC: Smooth muscle cell
TC: Total cholesterol
TG: Triglyceride
